# Simultaneous fabrication of multiple tablets within seconds using tomographic volumetric 3D printing

**DOI:** 10.1016/j.ijpx.2023.100166

**Published:** 2023-02-08

**Authors:** Lucía Rodríguez-Pombo, Laura Martínez-Castro, Xiaoyan Xu, Jun Jie Ong, Carlos Rial, Daniel Nieto García, Alejandro González-Santos, Julian Flores-González, Carmen Alvarez-Lorenzo, Abdul W. Basit, Alvaro Goyanes

**Affiliations:** a Departamento de Farmacología, Farmacia y Tecnología Farmacéutica, I+D Farma (GI-1645), Facultad de Farmacia, Instituto de Materiales (iMATUS) and Health Research Institute of Santiago de Compostela (IDIS), Universidade de Santiago de Compostela, 15782 Santiago de Compostela, Spain; b Department of Pharmaceutics, UCL School of Pharmacy, University College London, 29-39 Brunswick Square, London WC1N 1AX, UK; c FabRx Ltd., Henwood House, Henwood, Ashford, Kent TN24 8DH, UK; d Complex Tissue Regeneration Department, MERLIN Institute for Technology Inspired Regenerative Medicine, Universiteitssingel 40, 6229ER Maastricht, the Netherlands; e Facultad de Física, Centro de Investigación en Tecnologías Inteligentes (CITIUS), Universidade de Santiago de Compostela, 15782 Santiago de Compostela, Spain

**Keywords:** Vat photopolymerization 3D printed medicines, 3D rotary printing, Tomographic reconstruction, 3D printed drug products, Printing pharmaceuticals and drug delivery systems, Personalized formulations

## Abstract

3D printing is driving a shift in patient care away from a generalised model and towards personalised treatments. To complement fast-paced clinical environments, 3D printing technologies must provide sufficiently high throughputs for them to be feasibly implemented. Volumetric printing is an emerging 3D printing technology that affords such speeds, being capable of producing entire objects within seconds. In this study, for the first time, rotatory volumetric printing was used to simultaneously produce two torus- or cylinder-shaped paracetamol-loaded Printlets (3D printed tablets). Six resin formulations comprising paracetamol as the model drug, poly(ethylene glycol) diacrylate (PEGDA) 575 or 700 as photoreactive monomers, water and PEG 300 as non-reactive diluents, and lithium phenyl-2,4,6-trimethylbenzoylphosphinate (LAP) as the photoinitiator were investigated. Two printlets were successfully printed in 12 to 32 s and exhibited sustained drug release profiles. These results support the use of rotary volumetric printing for efficient and effective manufacturing of various personalised medicines at the same time. With the speed and precision it affords, rotatory volumetric printing has the potential to become one of the most promising alternative manufacturing technologies in the pharmaceutical industry.

## Highlights

• Tomographic volumetric 3D printing was used to fabricate paracetamol-loaded tablets.
• Torus- and cylinder-shaped tablets were printed.
• Two tablets were printed simultaneously within 12 to 32 s.
• Printed tablets exhibited pH-independent sustained drug release profiles.
• High-throughput pharmaceutical printing is feasible with this technology.

## Graphical abstract

**Figure:**
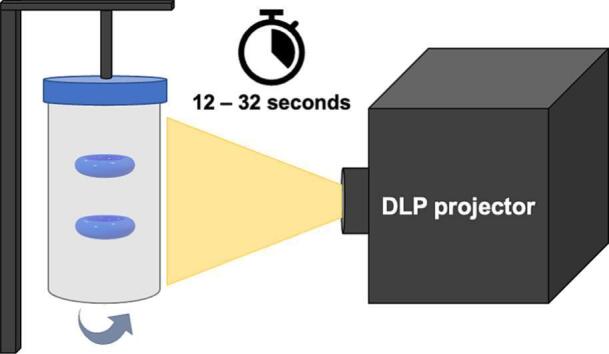

## 1 Introduction

In the pharmaceutical industry, 3D printing (3DP) is challenging conventional fixed-dose drug mass manufacturing systems by enabling tailored manufacturing (Andreadis et al., 2022; Awad et al., 2021; Hoffmann et al., 2022; Melocchi et al., 2021; Myung et al., 2022; Ragelle et al., 2021; Seoane-Viaño et al., 2021). It is possible to design personalised solid pharmaceutical forms (Krkobabic et al., 2020; Trenfield et al., 2020; Xu et al., 2021a; Xu et al., 2021c) by adjusting the dose and modifying the size or shape, which in turn can enhance therapeutic response (Dumpa et al., 2021; Goyanes et al., 2019; Trenfield et al., 2023; Wang et al., 2021). However, to be integrated into fast-paced clinical settings, 3D printers must afford high throughput and dimensional accuracy (Awad et al., 2022). Volumetric 3DP, or holographic printing, is a novel additive manufacturing technology that possess these attributes. Volumetric 3DP involves the simultaneous printing of the entire desired geometry by irradiating a resin-containing cuvette with a set of 2D images of the object viewed from different angles (Kelly et al., 2019; Zhang et al., 2021). In volumetric printing, all the points of the object photopolymerize and solidify at the same time inside the resin container. Consequently, 3D printed objects are fabricated in a matter of seconds, significantly faster than other layer-by-layer 3DP technologies. Apart from its speed, other advantages include the possibility of using high viscosity resins, the obviation of support structures, and the layerless structure of printed objects (Kelly et al., 2019; Madrid-Wolff et al., 2022; Shusteff et al., 2017).

Volumetric 3D printing has already been successfully used to produce 3D-printed Printlets loaded with paracetamol within 7 s (Rodríguez-Pombo et al., 2022). The volumetric printer used in that study employed a mirror-based system, wherein a system of mirrors split the light beam in three different directions, projecting 2D images from three different angles of the desired object on the same point. Localised regions where the three images are superimposed exceed the light dose threshold necessary to induce photopolymerization, inducing the creation of the 3D structure in a single step (Kelly et al., 2019; Shusteff et al., 2017). Despite its speed, there are two limitations to that configuration of volumetric 3D printing: only one object has been printed at a time so far, and only objects with a plane of symmetry can be printed since only three viewing angles are appreciated. An alternative system design is needed to improve the versatility and throughput of volumetric 3D printing.

An improved form of volumetric 3D printing is built upon the concept of computerized axial tomography, a technology widely used for diagnostic imaging in medicine (Kelly et al., 2019). Computed Tomography (CT) is a radiological imaging method that has been extensively used for medical diagnosis since 1970 (Ter-Pogossian, 1977). This technique provides different 2D images of transverse sections of the body's tissues or organs due to the variations in the attenuation of radiation in the 3D object imaged. Therefore, the anatomical structure can be reconstructed from projections taken at various angles around the object and applying suitable algorithms (Ter-Pogossian, 1977). Tomographic volumetric printing (also known as tomographic reconstruction or rotary volumetric printing) is inspired by CT scan, but in reverse. In essence, a cylindrical container filled with photosensitive resin is set into rotation while it is being irradiated from one side with computed patterns of light (Bernal et al., 2022; Bernal et al., 2019; Gehlen et al., 2022; Kollep et al., 2022; Loterie et al., 2020; Toombs et al., 2022). These light patterns are produced by a digital light processing (DLP) projector, and they are displayed in sync with the rotational movement of the resin container. The patterns represent 2D images of the object viewed at distinct rotational angles and they are computed by a Radon transform similarly to CT-scan (Ter-Pogossian, 1977). The light patterns projecting from a single angle are not sufficient to solidify a 3D object from the photosensitive resin completely. However, if the container is irradiated from every angle by all the 2D images, a 3D distribution of accumulated light dose is created (Kelly et al., 2017; Loterie et al., 2020). When a gelation threshold is reached, the illuminated liquid resin polymerizes by a chain reaction into a solid polymer (Zissi et al., 1996). Tomographic volumetric printing could further accelerate printing times by enabling the fabrication of multiple objects simultaneously. It also enables the printing of objects with greater structural complexities as all viewing angles of the object can be projected.

Therefore, the main objective of this work is to evaluate, for the first time, the use of rotary volumetric printing for the fabrication of oral pharmaceutical dosage forms. Two different shapes were designed, torus and cylinder, with the intention of simultaneously printing two of these shapes during the same printing process. We studied and optimized the critical parameters of the printing process (rotation speed, light intensity, and exposure time) for six different formulations with paracetamol loaded at 5% *w*/w. Finally, physicochemical characterization techniques were used to evaluate the properties of the objects printed by this new technology. The drug content was determined and the release profiles of both torus and cylinder shapes were compared for the different printed formulations.

## 2 Materials and methods

### 2.1 Materials

Paracetamol (MW 151.16 g/mol) was used as the model drug. Polyethylene glycol diacrylate (PEGDA) of different average molecular weights (PEGDA MW 575 g/mol and 700 g/mol) was used as the photoreactive monomer. Polyethylene glycol (PEG) with an average molecular weight of 300 g/mol (PEG 300 g/mol) and distilled water were used as the diluents. Lithium phenyl-2,4,6-trimethylbenzoylphosphinate (LAP, MW 294.21 g/mol, ≥ 95%) was used as the photoinitiator. Isopropanol (MW 60.1 g/mol, 99.8% Ph. Eur.) was used to wash the printed objects. Acetonitrile (≥ 99.9%, HPLC grade) and methanol (HPLC grade) were used as mobile phase for drug content assay. All materials were purchased from Sigma-Aldrich (Dorset, UK). Sodium phosphate tribasic dodecahydrate (≥ 98.0%) was purchased from Honeywell Fluka (Buchs, Switzerland). Hydrochloric acid (37%, Ph. Eur) was purchased from Scharlau (Barcelona, Spain). All materials were used as received.

### 2.2 Preparation of drug-loaded resins

Six different formulations with different compositions were tested (Table 1). 50 g of resin were prepared for each of the formulations. All the components were weighed using an analytical balance and placed in an amber glass bottle to protect them from light, and the resulting solution was left stirring at 200 rpm on a multiposition shaker plate (Ovan, Spain) at room temperature until complete dissolution (2−10*h*). Resins were used immediately after preparation.

**Table 1:** Compositions of the formulations tested in rotary volumetric printing.

Formulation	PEGDA 575 (% w/w)	Water (%w/w)	PEGDA 700 (% w/w)	PEG 300 (% w/w)	LAP (% w/w)	Paracetamol (% w/w)
PW35–65	33.241	61.734	–	–	0.025	5
PW65–35	61.734	33.241	–	–	0.025	5
PW90–10	85.478	9.497	–	–	0.025	5
PP35–65	–	–	33.241	61.734	0.025	5
PP65–35	–	–	61.734	33.241	0.025	5
PP90–10	–	–	85.478	9.497	0.025	5

### 2.3 Absorbance characterization of photosensitive resins

Once the photosensitive resins were prepared, the absorbance values of the resins were recorded in a spectrophotometer (Agilent 8453, Germany) at two wavelengths: 385 nm and 405 nm in a 10 mm light path cuvette.

### 2.4 3D design and printing process

**Fig. 1:**
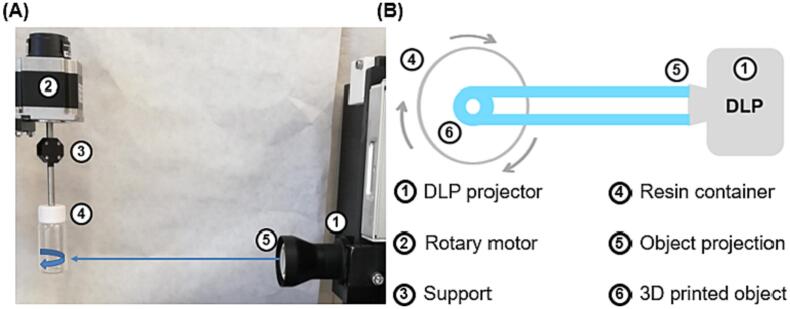
(A) Photograph of the rotary volumetric printer and (B) Schematic illustrating the printing process using the rotary volumetric printer (top view). Legends corresponding to each component of the system are numbered on the right side.

The volumetric printer (FabRx Ltd., United Kingdom) consisted of a digital light projector (Wintech DLP6500, USA), which emitted UV light at a wavelength of 385 nm in the direction of the rotating resin container (Fig. 1). The cylindrical resin container (2.5 cm diameter x 5 cm height) was suspended by an axis attached to a motor that allowed 360° rotation. The resin container was located at a distance of 23 cm from the light source (DLP projector) and at a height of 15 cm from the base.

**Fig. 2:**
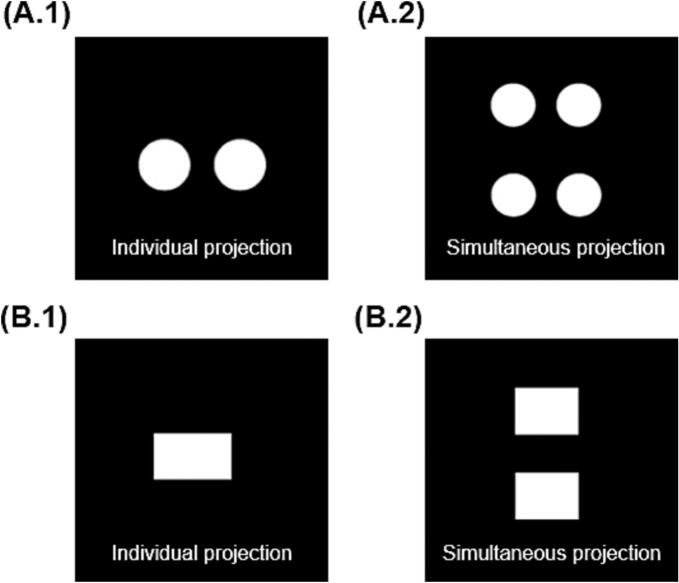
Projection images of (A.1) torus shape and (B.2) cylinder shape individual projection for single printlet for preliminary tests and (A.2) torus shape and (B.2) cylinder shape simultaneous projection for multiple Printlets.

For this study, torus and cylinder shapes were printed, which were fabricated using the projections shown in Fig. 2. The torus dimensions were 16 mm diameter x 7 mm height x 2 mm inner diameter (Fig. 2A.1) and cylinder dimensions were 14 mm diameter x 10 mm height (Fig. 2B.1). These geometric figures were created using Microsoft Paint (Version 6.3, build 9600), exported in .jpg format, and loaded into a software designed by FabRx (London, UK) that controls the printer. The software projects the image of the object in two dimensions on the resin container, which coupled by the rotating motion of the resin container results in the desired 3D structure. Specifically, by projecting an image of two circles or a rectangle, the shape of a torus or a cylinder was obtained, respectively (Fig. 2A.2 and 2B.2). In addition, two Printlets could be created at the same time in the same printing process by projecting one on top of the other (Fig. 2A.2 and 2B.2, right hand side).

The photosensitive resin (as prepared in Section 2.2) was introduced into the container, which was then attached to the rotary motor via the axis support. The container was carefully positioned at the appropriate height to ensure that the light beam from the projector aligned with the centre of the container. To start printing, the resin container was rotated at 30 rpm, and then the image was projected for a variable period of time, depending on the formulation. Two objects were printed simultaneously, and the printing process was repeated several times as needed.

After printing, the Printlets were removed from the cuvette and washed with isopropanol for 10 s to eliminate any unpolymerized monomers on the surface. Then, the Printlets were post-cured by placing them in an oven (Heraeus I42, Germany) at 20 °C for 1 h (30 min on one side of the object and 30 min on the other side) under UV light (wavelength 375 nm) from an ultraviolet lamp (Philips BLB F8 T5, The Netherlands). Finally, the Printlets were stored in individual vials to protect them from light and moisture.

### 2.5 Physicochemical characterization of resins and Printlets

#### 2.5.1 Environmental scanning electron microscopy (ESEM)

Printlets were cut in half and attached onto a self-adhesive carbon disc mounted on a 25 mm aluminium stub, which was coated with 25 nm of gold using a sputter coater. The stub was then placed into a FEI Quanta 200 FEG Scanning Electron Microscope (FEI, UK) at 5 kV accelerating voltage using secondary electron detection to obtain the cross-section images of the Printlets.

#### 2.5.2 Thermal analysis

Differential scanning calorimetry (DSC) was used to characterize the thermal behaviour of the 3D printed formulations. DSC measurements were carried out, in duplicate, using a DSC Q200 (TA Instruments, New Castle, DE, USA) with a refrigerated cooling accessory at a heating rate of 10 °C/min. The calorimeter was calibrated for baseline using no pans, for cell constant and temperature using indium (melting point 156.61 °C, enthalpy of fusion 28.71 J/g), and for heat capacity using sapphire standards. The range of the temperature was 0–200 °C and nitrogen was used as the purge gas at a flow rate of 50 mL/min. All experiments were performed using non-hermetic aluminium pans, in which 3–5 mg of blends were accurately weighed, and then covered with the lid. Data were collected with TA Advantage software for Q series (version 2.8.394) and analyzed using TA Instruments Universal Analysis 2000. All melting temperatures were reported as extrapolated peak unless otherwise stated.

#### 2.5.3 X-ray powder diffraction (XRPD)

X-ray powder diffraction patterns were obtained in a D8 Advance (Bruker, Billerica, MA, USA) using the Bragg-Brentano focusing geometry, equipped with a sealed X-ray tube ((CuKα1 (λ = 1.5406 Å)) and a LYNXEYE-type detector. The intensity and voltage applied were operating at 40 mA and 40 kV, respectively. The diffractograms were obtained in the 2θ angular range of 3–60^0^ with a step of 0.02° and a counting time of 2 s per step. Samples were deposited on an oriented Si(511) plate to avoid scattering noise caused by a glass support. Samples were rotated during measurement to obtain optimal peak profiles for analysis and to minimize the effect of the preferential orientation. Mathematical analysis of the obtained diffractograms was performed using the HighScore Plus (version 3.0d) software.

#### 2.5.4 X-ray micro computed tomography (micro-CT)

To visualize the internal structure of the Printlets, a high-resolution X-ray micro computed tomography (Micro-CT) scanner (SkyScan1172, Bruker-microCT, Kontich, Belgium) was used. Each image was obtained by rotating the Printlet through 180° with a frame averaging of 4 and a 0.5° rotation step using medium camera resolution (2000 × 1048 pixels). Image reconstruction was performed using NRecon software (Version 1.7.0.4, Bruker-microCT, Kontich, Belgium) and the reconstructed images were processed and visualized using the Ctan software (version 1.15.4).

#### 2.5.5 Fourier-transform infrared spectroscopy (FTIR)

The infrared spectra of paracetamol, photosensitive resins and Printlets were collected using a Spectrum 100 FTIR spectrometer (PerkinElmer, Waltham, MA). All samples were scanned between 4000 and 650 cm^−1^ at a resolution of 4 cm^−1^ resolution for 6 scans.

### 2.6 Drug content in the Printlets

Paracetamol-loaded Printlets prepared using the volumetric printer were crushed into fine particles using a mortar and a pestle. 25 mL of acetonitrile were gradually added to the mortar during the crushing process. The mixture was transferred to a 1 L volumetric flask and Milli-Q water was added. The mixture was then subjected to magnetic stirring (200 rpm) overnight. Finally, samples of solution were filtered through 0.22 μm filters (Millipore Ltd., Ireland) and the concentration of drug was determined using HPLC. JASCO LC-4000 Series HPLC system (Jasco, Spain) equipped with a degasser, quaternary pump, column heater, autosampler and UV/Vis detector, was used. 20 μL of sample was injected into a Waters Spherisorb 5 μm C8 column, 4.6 mm × 250 mm (Waters, Milford, MA, USA). The compounds were separated using a mobile phase composed of water (85% *v*/v) and methanol (15% v/v), which was pumped at a flow rate of 1 mL/min. The temperature was maintained at 40 °C and the eluents were assessed at a wavelength of 247 nm. The retention time was 8 min. All measurements were made in duplicate. To quantify the drug concentration based on the HPLC results, standard solutions of paracetamol ranging from 0.002 to 0.02 mg/mL were prepared and analyzed using HPLC. The resulting calibration curve (R^2^ = 0.9999) was used to quantify the drug concentration in the Printlets.

### 2.7 Water uptake

Tori derived from each formulation were evaluated in duplicate. The initial weight (Wo) was measured before immersing them in distilled water (50 mL). After 48 h, the final weight (Wf) of the tori were measured after gently removing the excess liquid on the surface with filter paper. The water uptake was calculated using the following equation:

$$\text{Water uptake}\;\left(\%\right)=\frac{\mathrm{Wf}-\mathrm{Wo}}{\mathrm{Wo}}*100$$

In addition, the dimensions (diameter x height x inner diameter) were measured again.

### 2.8 Mechanical properties

Tori derived from each formulation were evaluated, in duplicate, using a TA.XT Plus Texture Analyzer (Stable Micro Systems, Surrey, UK) equipped with a 30 kgf (∼294 N) load cell. The samples were subjected to 5 successive stress-strain cycles (pre-assay and post-assay speed were 0.50 mm/s), applying a uniaxial compression along their short axis (height) by downward movement (0.50 mm/s) of an aluminium cylinder probe (20 mm in diameter). The activation strength was set at 0.0010 N. In each cycle, the torus was compressed until a force of 2 kgf (19.6 N) was reached, and the force-displacement data were recorded for each compression cycle and later converted to engineered stress and strain, using the initial dimensions of the torus. The Young's modulus was calculated as the slope of the initial linear region of the stress-strain curves. The area under the stress-strain curve was calculated in each of the 5 cycles. Samples of the torus were immersed in distilled water (50 mL) for 48 h and their mechanical properties were subsequently evaluated again.

### 2.9 In vitro release studies

The drug release profiles of the Printlets were evaluated using a SR8-Plus Dissolution Test Station (Hanson Research, Chatsworth, CA, USA) with USP-II apparatus. The Dissolution Test Station was connected to a pump system Auto Plus DissoScan (Hanson Research, Chatsworth, CA, USA). The speed of the paddles was set at 50 rpm with a temperature of 37 ± 0.5 °C. The Printlets were dropped in 750 mL of 0.1 M HCl for 2 h to simulate gastric conditions. After 2 h, 250 mL of trisodium phosphate solution (0.2 M) was added into each vessel and the pH was adjusted to 6.8 using 1 M NaOH or 1 M HCl solutions to simulate intestinal conditions. During the dissolution assay, samples were automatically withdrawn and filtered through 10 μm filters. The concentration of paracetamol in each sample solution was determined using an in-line UV spectrophotometer (Agilent 8453, Germany) at a wavelength of 243 nm. At the end of the assay, 1 mL of sample was withdrawn from each vessel, filtered through 0.22 μm filters (Millipore Ltd., Ireland), and analyzed using HPLC to determine the final amount of drug released. Tests were conducted in triplicate under sink conditions. Data were reported throughout as mean ± standard deviation (*n* = 3).

## 3 Results and discussion

### 3.1 Rotary volumetric printing process

PEGDA 575 and 700 monomers were selected for their photoreactivity and crosslinking ability (Wang et al., 2016). Water and PEG 300 were used as diluents to modulate paracetamol release. While the presence of water might affect the long-term stability of the loaded drug in the printed dosage form, this issue is mitigated by the intended application of the technology: as Printlets will be fabricated on-demand, long-term storage is not expected. LAP was the chosen photoinitiator because of its strong absorption at a wavelength of 385 nm (wavelength emitted by the digital light projector), its high solubility in water, and its initiation efficiency (Qin et al., 2018). Although the employed photoreactive monomers and the photoinitiator have yet to be approved for the pharmaceutical use, there are other materials (dental resin composites) that are commonly used in dentistry (Aminoroaya et al., 2021). For all six resin formulations, paracetamol was dissolved completely, and the final resins showed a transparent appearance. Transparency of the resin is critical in ensuring that tomographic volumetric printing proceeds accurately, since turbidity in the resin may cause scattering of the light emitted by the projector and results in inaccurate or failed prints (Kitchener et al., 2017; Sánchez Soler and Espías Gómez, 2004).

Initially, different critical parameters were modified empirically to determine what effects and importance they had on the final 3D printed object. It was observed that the size and weight of the object increased with an increase in exposure time, light intensity, and rotation speed. After this preliminary study, the adequate values of rotation speed and light intensity for the respective shapes were investigated. This was based on preliminary tests for each formulation, considering the movement of the resin with respect to the projector. An intensity value corresponding to 62.75% of the total brightness of the projector (corresponding to an intensity factor of 160 in the software) was found optimal to achieve adequate levels of crosslinking for all formulations, i.e. it did not induce overcuring (large prints occupying the entire diameter of the vial) nor did it result in incomplete objects. Likewise, a rotation speed of 30 rpm was found to be the most suitable for printing both shapes. To accommodate for different rates of photopolymerization in different formulations, only the exposure time was adjusted (i.e., the rotation speed and light intensity were kept constant at 30 rpm and 62.75%, respectively).

**Fig. 3:**
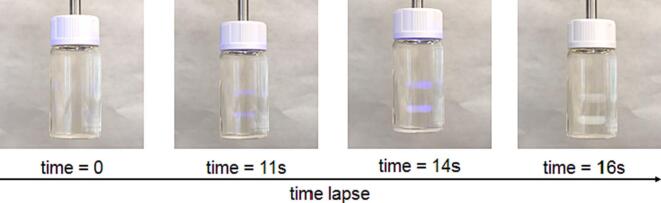
Picture of the sequential view of the resin container during the double Printlet fabrication process (corresponding to PW 35–65 torus).

Printing was completed between 12.5 and 32 s, with PW65–35 printing the fastest and PP90–10 printing the slowest. The sequence of the simultaneous printing process of torus is showed in the Fig. 3 and in Supplementary Video 1.

**Fig. 4:**
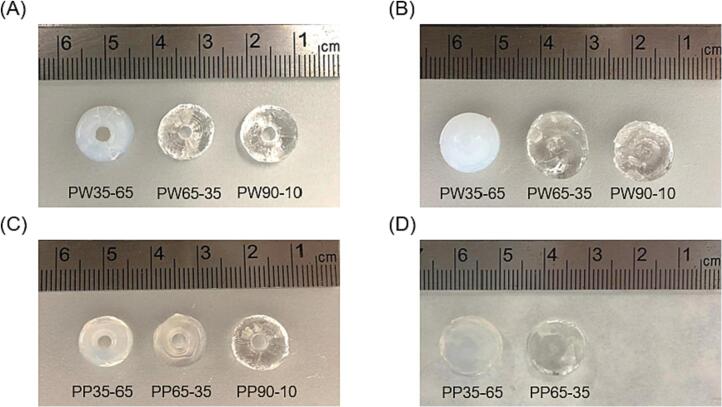
Photographs of the torus and cylinder Printlets obtained with different formulations. (A) From left to right: Torus of PW35–65, PW65–35 and PW90–10 formulations. (B) From left to right: Cylinder of PW35–65, PW65–35 and PW90–10 formulations. (C) From left to right: Torus of PP35–65, PP65–35 and PP90–10 formulations. (D) From left to right: Cylinder of PP35–65 and PP65–35 formulations.

Both shapes were successfully printed for the remaining formulations. Fig. 4 shows the torus and cylinder shapes successfully printed with each formulation:

All the obtained Printlets, regardless of their composition and the chosen shape, showed uniformity in weight and size as shown in Table 2. The measurements obtained for the torus shape were 9.96 ± 0.2 mm in diameter and 3.94 ± 0.22 mm in height. The dimensions of the cylinders were 11 ± 0.18 mm in diameter and 4.96 ± 0.17 mm in height. These are slightly smaller than those of the projections used (16 × 7 mm and 14 × 10 mm, respectively for torus and cylinder) since the cuvette that contained the resin had a convex surface, which caused inconsistent refraction of light rays. This caused the image projected inside the resin container to be smaller than the original programmed image, and therefore a smaller than expected printed object. Apart from the resin's parameters (viscosity and reactivity) and light dose distribution, the resolution of the 3D printed object and its fidelity to the digital model depends on the etendue of the illumination patterns (Loterie et al., 2020). As we described above, the resin container was cylindrical. Consequently, light strikes the resin, which has a different refractive index from air, at different angles, causing the projection to be distorted. A possible solution to maintain the fidelity to the torus/cylinder dimensions could be used a rectangular prism first (flat sides) filled with a fluid of the same refractive index as the photosensitive resin (without the photoinitiator). Then, the convex container would be submerged in this first cuvette with rectangular sides. This can avoid distortions of the incident projection due to the cylindrical lensing effects (Kelly et al., 2017; Loterie et al., 2020).

**Table 2:** Exposure time, and main characteristics of each formulation. The results are shown as mean ± standard deviation.

Formulation	Shape	Exposure time (s)	Diameter (mm)	Inner diameter (mm)	Height (mm)	Weight (g)
PW35–65	Torus	16	9.90 ± 0.29	4.80 ± 0.42	3.92 ± 0.25	0.452 ± 0.091
	Cylinder	13.5	11.03 ± 0.14	–	4.88 ± 0.16	0.847 ± 0.072
PW65–35	Torus	12.5	10.01 ± 0.17	4.60 ± 0.84	3.90 ± 0.26	0.462 ± 0.089
	Cylinder	12.5	10.95 ± 0.23	–	4.97 ± 0.15	0.901 ± 0.084
PW90–10	Torus	20	9.99 ± 0.21	4.90 ± 0.32	3.92 ± 0.19	0.419 ± 0.036
	Cylinder	18	10.97 ± 0.15	–	4.98 ± 0.20	0.771 ± 0.064
PP35–65	Torus	26	10.03 ± 0.12	4.30 ± 0.95	4.00 ± 0.13	0.450 ± 0.042
	Cylinder	28	11.07 ± 0.22	–	5.02 ± 0.13	0.922 ± 0.041
PP65–35	Torus	25	9.86 ± 0.23	4.90 ± 0.32	3.90 ± 0.25	0.359 ± 0.047
	Cylinder	27	10.98 ± 0.16	–	4.93 ± 0.22	0.921 ± 0.083
PP90–10	Torus	32	9.96 ± 0.18	4.70 ± 0.67	4.02 ± 0.24	0.370 ± 0.020
	Cylinder	–	–	–	–	–

The exposure times shown in Table 2 reflect the time needed to print two objects, in this case. It is important to note that the overall exposure time is unlikely to change with the number of objects being printed simultaneously, i.e. if given a larger cuvette and larger light projection, 100 objects can be printed at the same time. Therefore, this technology is highly amenable to high-throughput production of large quantities of Printlets.

It was not possible to print cylinders with dimensional accuracy using the PP90–10 formulation. Elongated cylinders with incomplete bottoms were obtained from this formulation as the objects moved upwards during the photopolymerization process. This could be due to the relatively higher monomer content resulting in the solidified matrix possessing a lower density, which when coupled with other physical forces such as centripetal force caused the object to move.

### 3.2 Characterization of resins and Printlets

#### 3.2.1 ESEM

**Fig. 5:**
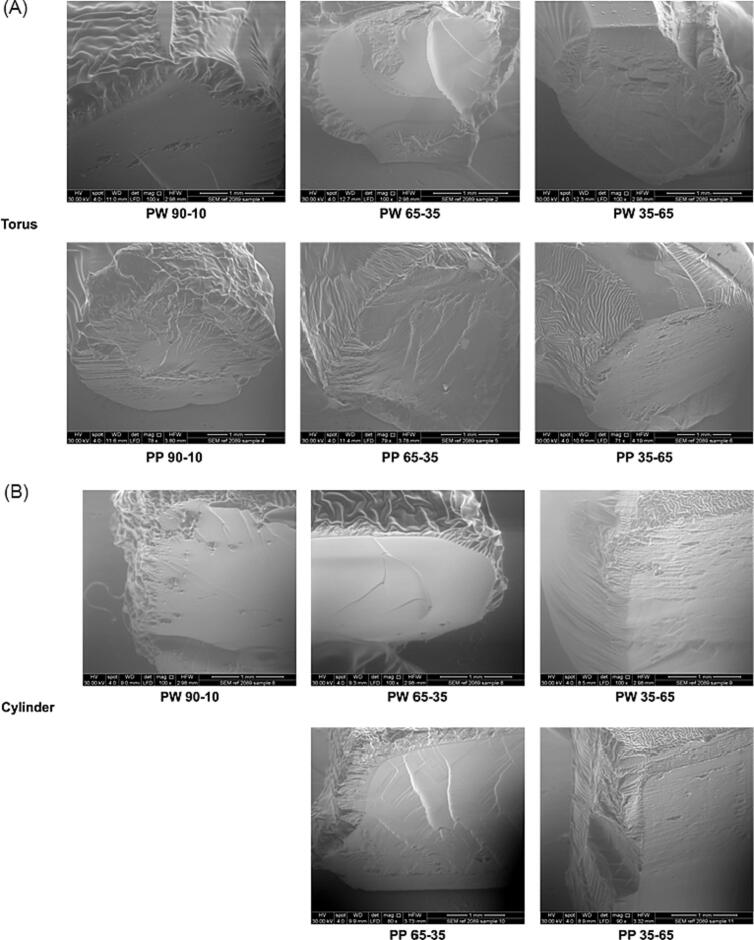
ESEM images of (A) torus shape and (B) cylinder shape Printlets. The scale bar is equivalent to 1 mm.

The images obtained from ESEM microscopy allowed observation of the surface and the cross-sections of the torus and cylinder Printlets (Fig. 5A and B, respectively). The printed objects showed uniform and layer-less surfaces. This indicated the absence of crystallized drug and showed that the 3D structures were created in a single step, unlike other layer-based additive manufacturing processes.

**Fig. 6:**
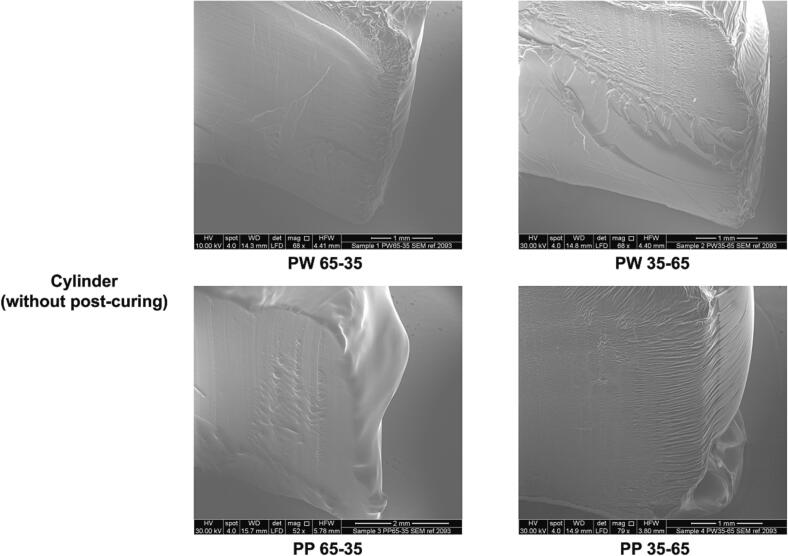
ESEM images of PW65–35, PW35–65, PP65–35 and PP35–65 cylinder Printlets without post-curing process in the UV oven. The scale bar is equivalent to 1 mm.

A “core-shell” structure could be observed for all Printlets. This can be attributed to the post-curing process since all objects were subjected to UV radiation for 1 h in the oven (described in Section 2.4). The interior appeared smoother than the surface because the surface was more exposed to UV radiation than the core since the UV radiation was attenuated as it penetrated through the object. Consequently, a greater degree of photopolymerization occurred on the surface of the objects during post-curing, resulting in a shell-like appearance. To investigate the hypothesis that the observed “core-shell” structure in the Printlets was due to the post-curing process, ESEM images of PW65–35, PW35–65, PP65–35 and PP35–65 cylinders that were not subjected to post-curing nor washing were obtained (Fig. 6). As the images show, the surface and the cross-section are completely homogeneous and smooth. Compared to Fig. 5, the “core-shell” structure is not observed, strongly suggesting that the “core-shell” structure was due to UV radiation during post-curing.

For the cylinders (Fig. 5B), homogeneous surfaces and a smooth interior were generally observed, although objects derived from PW35–65 and PP35–65 formulations exhibited a slightly greater degree of surface heterogeneity. This is likely due to a lower proportion of light absorbing monomer (PEGDA with the reactive double bonds) and more hydrophilic less light absorbing agents (water and PEG 300), allowing UV light to penetrate deeper into the 3D structures during post-curing in an oven. For the torus (Fig. 5A), these differences were more appreciable, since a higher percentage of surface area was exposed to UV radiation.

#### 3.2.2 X-ray micro computed tomography (micro-CT)

**Fig. 7:**
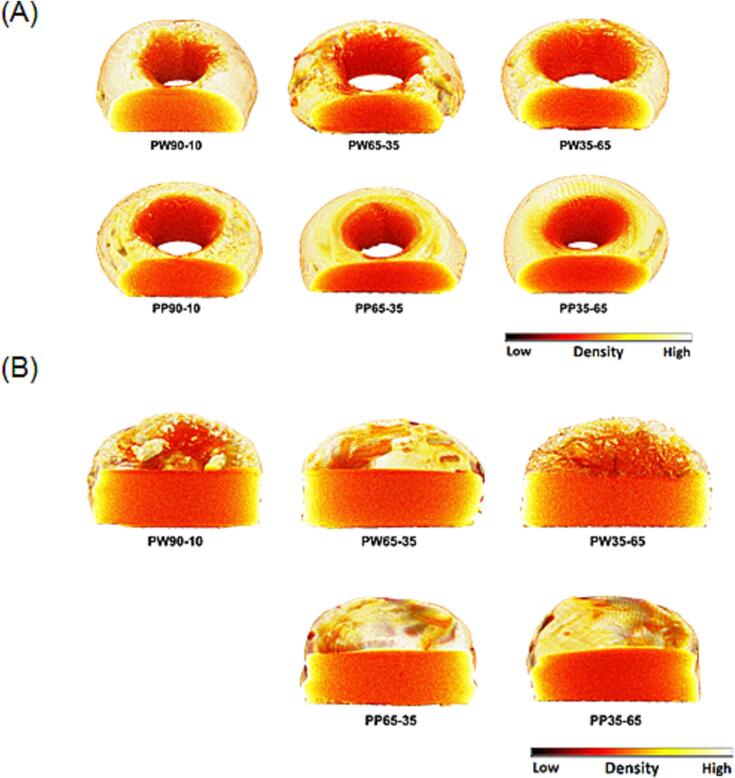
X-ray micro-CT images of different A) cylinder shape and B) torus shape Printlets. The colour scale bar represent density.

X-ray micro-CT imaging was used to visualize the internal structure of the Printlets and study their density (Fig. 7). Both the torus and cylinder Printlets showed an internal structure that was less dense in the core and denser on the surface. This corroborates with the observations made from the ESEM images in the previous section, whereby post-curing under UV-irradiation induced a higher degree of photopolymerization on the surface of the objects compared to the internal core. The higher extent of photopolymerization in turn resulted in higher structural density at the surface of the objects. Nonetheless, there were no perceivable differences in the pattern of structural density between the different formulations nor between the torus and cylinder shapes, indicating that the chosen parameters for each formulation and/or object resulted in similar degrees of polymerization. Interestingly, while pores were expected to be present due to the removal of water and PEG, these were not observable in the micro-CT images and the ESEM images. Stronger magnification might be required to observe these structural features, especially for Printlets with a relatively lower proportion of non-reactive diluent.

#### 3.2.3 DSC and XRPD

**Fig. 8:**
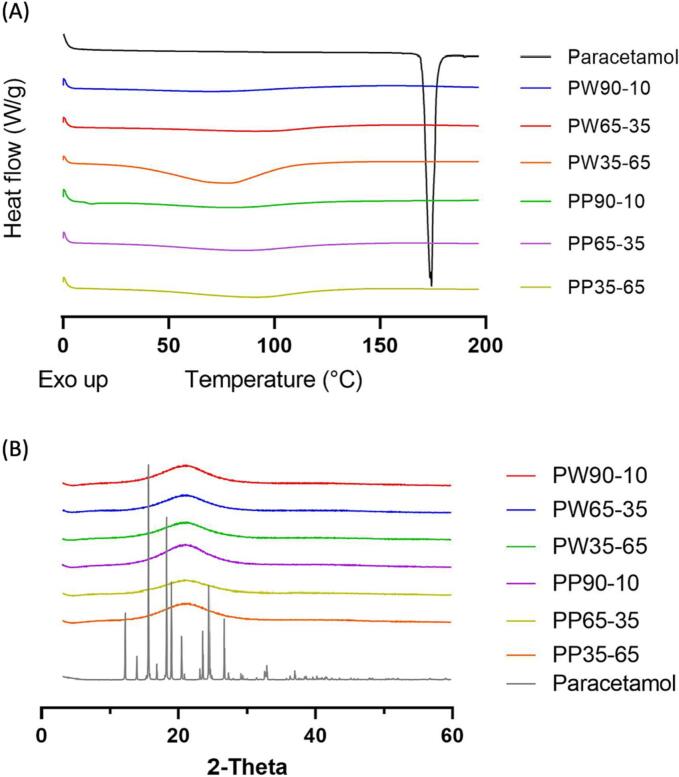
(A) DSC thermograms of pure paracetamol and different Printlets. (B) X-ray powder diffractograms of pure paracetamol and different Printlets.

DSC and XRPD were performed to determine the physical status of paracetamol in the Printlets. Pure paracetamol had a melting point at 169 °C (Fig. 8A). The absence of this endothermic peak in the DSC thermograms of the Printlets suggests that paracetamol was completely dissolved in the resin formulations and was molecularly dispersed in the obtained Printlets. The broad endotherm at 100 °C shown in PW35–65 can be attributed to water evaporation upon heating. XRPD results (Fig. 8B) also confirmed the absence of crystallized drug in the Printlets since no paracetamol crystallization peaks were found in the diffractograms of all Printlets.

#### 3.2.4 Drug content in the Printlets and FTIR

The drug loading of each formulation is reported in Table 3. As in previous studies, the actual drug load was slightly lower than the theoretical load of 5% *w*/w.

**Table 3:** Drug loading results and Young's modulus results before and after water uptake assay (denoted as “initial” and “final”, respectively) of paracetamol-loaded Printlets. Results are shown as mean ± standard deviation.

Printlet	Drug loading (%)	Theoretical drug load (%)	Initial Young's modulus (MPa)	Final Young's modulus (MPa)
PW35–65	4.56 ± 0.22	91.2 ± 0.04	2.37 ± 0.21	2.48 ± 0.07
PW65–35	4.57 ± 0.14	91.4 ± 0.03	2.08 ± 0.77	1.73 ± 0.24
PW90–10	4.55 ± 0.06	91.0 ± 0.01	3.04 ± 0.40	2.22 ± 0.04
PP35–65	4.61 ± 0.03	92.2 ± 0.01	1.26 ± 0.01	1.03 ± 0.06
PP65–35	4.54 ± 0.03	90.8 ± 0.01	2.32 ± 0.22	1.37 ± 0.25
PP90–10	4.61 ± 0.03	92.2 ± 0.01	1.69 ± 0.28	2.00 ± 0.59

**Fig. 9:**
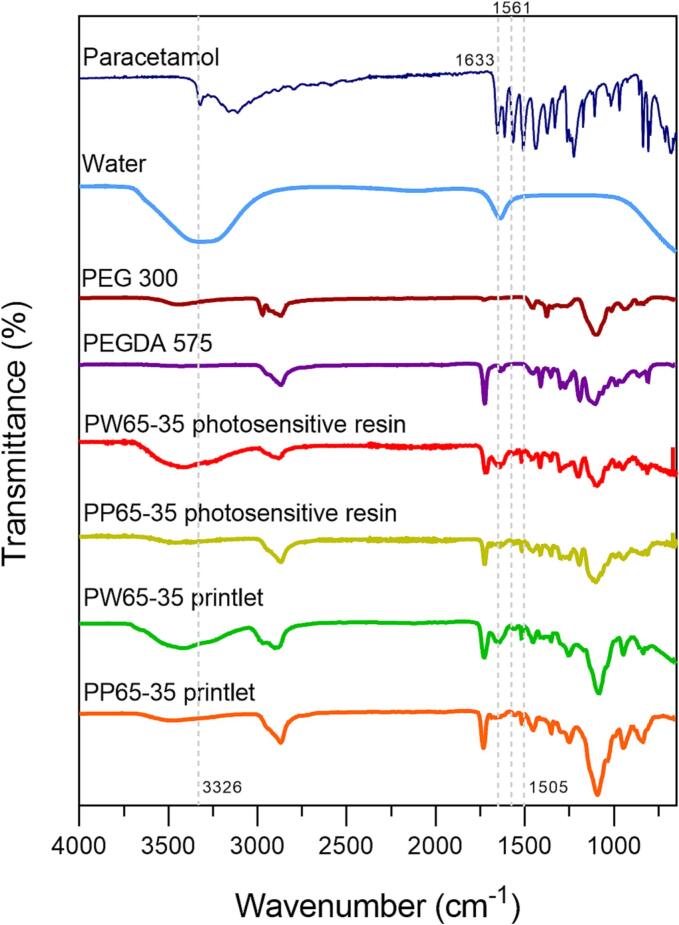
FTIR spectra of pure paracetamol, water, PEG 300, PEGDA 575, PW 65–35 and PP 65–35 photosensitive resin, PW 65–35 and PP65–35 Printlets.

FTIR was performed to investigate any possible chemical interactions between paracetamol and the monomer (PEGDA) during the printing process (Fig. 9). In the spectrum of paracetamol powder, the peaks at wavelengths of 3326 cm^−1^, 1561 cm^−1^ and 1505 cm^−1^ corresponded to O—H, N—H amide and C—H bonds, respectively (Krkobabic et al., 2019; Mk, 2015). Apart from the peak at 3326 cm^−1^, the 1561 cm^−1^ and 1505 cm^−1^ absorption bands were clearly observed in the spectra of PW65–35 and PP65–35 photosensitive resins and Printlets, indicating that there were no detectable covalent interactions between the paracetamol and the photopolymers. The peak at 3326 cm^−1^ was not detected in the spectra of the photosensitive resins since the broader peaks of water and PEG 300 that occurred at those same wavelengths covered it.

The spectrum of PEGDA 575 was used as a reference to show a peak at 1722 cm^−1^ corresponding to a C=O double bond and another peak at 1633 cm^−1^ for a C=C double bond. The decrease in the second band at 1633 cm^−1^ confirmed that the photopolymerization of the monomer took place, since the conversion of the double bond (C=C) to a single bond (C—C) attenuated the vibrational band in the case of the spectra of the PW65–35 and PP65–35 photosensitive resins (Kadry et al., 2019). Therefore, chemical interactions with PEGDA did not explain the observed negative deviation in drug loading. Moreover, it was previously demonstrated that there is no photodegradation of paracetamol during UV exposure (Rodríguez-Pombo et al., 2022). Instead, one possible reason could be an incomplete drug extraction from the polymer matrix (Wang et al., 2016). This is turn could be due to an equilibrium being reached between the paracetamol concentration in the extraction medium and that within the polymer matrix at the end of the extraction process, thus preventing any further diffusion of paracetamol out of the polymer matrix. Non-covalent interactions between the drug and polymer could also prevent complete drug extraction. Another reason could be the diffusion of paracetamol out of the matrix when the Printlets were briefly washed with isopropanol, given the relatively high solubility of paracetamol in isopropanol (Hojjati and Rohani, 2006).

#### 3.2.5 Mechanical properties and water uptake

Initially, torus were compressed until a 10 kgf was reached. However, only PW65–35 torus samples resisted those conditions without breaking. Therefore, the force was reduced to 2 kgf and none of the samples broke during the mechanical properties assay. One PP65–35 torus sample (out of two) broke in several smaller pieces after five consecutive-compression cycles, but it was not crushed. After immersion in water, the mechanical properties were evaluated again. All the Printlets (except PP90–10) were able to tolerate the compression force used in the assay. PP90–10 torus samples fractured into several smaller pieces without being crushed. Young's modulus was calculated for each sample as described in Section 2.8 and the results are summarised in Table 3.

Young's modulus indicates the linear relationship between the applied stress and the strain produced in the elastic zone. In this zone, the material recovers its initial shape when the compressive force is removed. Therefore, this paremeter indicates the rigidity or the deformation capacity of the material, wherein a higher Young's modulus indicates higher material rigidity. The Young's modulus of the Printlets ranged from 1.26 ± 0.01 MPa (PP35–65) to 3.04 ± 0.40 MPa (PW90–10) (Table 3). Interestingly, the Young's modulus decreased after immersion in water, with the exception of PW35–65 and PP90–10 formulations.

**Fig. 10:**
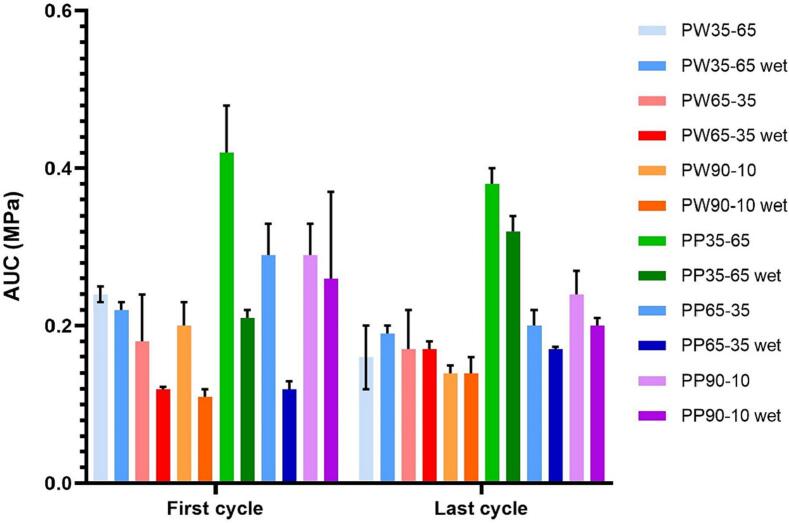
Graph with the values of the area under the stress-strain curve (AUC) in the first compression cycle and in the last compression cycle for Printlets before and after water uptake assay (named as “wet”).

The area under the stress-strain curve (AUC) results are shown in Fig. 10. The higher the value of the area under the curve, the greater the energy absorption capacity before deforming. As shown in Fig. 10, the AUC obtained during the first compression cycle is generally higher than that of the last compression cycle, except for PW65–35 wet, PW90–10 wet, PP35–65 wet, and PP65–35 wet. PEGDA 575-Water (PW) Printlets also generally exhibited lower AUC values than the equivalent PEGDA 700-PEG (PP) Printlets. Additionally, AUC values of each formulation were generally reduced after water uptake assay.

The results obtained after the immersion of the torus in distilled water are shown in Table 4. The solvent uptake induced dimensional changes (expansions) in diameter, height, and inner diameter, except in the case of PW35–65 formulation since initial and final measurements were the same. Generally, the Printlets swelled slightly after being immersed in distilled water. The presence of polar groups in the chemical structure of PEGDA allows water molecules to form hydrogen bonds with the cross-linked polymer network, inducing network expansion. The drug release mechanism could be influenced by mainly diffusion and, in part, by swelling since all the Printlets absorbed some water.

**Table 4:** Dimensions (before and after immersion) and water uptake of each formulation. The results are shown as mean ± standard deviation. *d*_*o*_ refers to the outer diameter, *h* refers to the height, and *d*_*i*_ refers to the inner diameter.

Formulation	Initial dimensions (mm)			Final dimensions (mm)			Water uptake (%)
	*d*_*o*_	*h*	*d*_*i*_	*d*_*o*_	*h*	*d*_*i*_	
PW35–65	11.0 ± 0.0	4.0 ± 0.0	5.0 ± 0.0	11.0 ± 0.0	4.0 ± 0.0	5.0 ± 0.0	11.0 ± 2.3
PW65–35	11.0 ± 0.0	3.0 ± 0.0	3.0 ± 0.0	14.0 ± 0.0	4.5 ± 2.1	4.0 ± 1.4	39.0 ± 1.3
PW90–10	11.0 ± 0.0	3.5 ± 0.7	4.5 ± 0.7	13.0 ± 0.0	4.5 ± 0.7	5.0 ± 0.0	29.0 ± 1.5
PP35–65	11.0 ± 0.0	3.0 ± 0.0	4.0 ± 1.4	14.0 ± 0.0	4.5 ± 0.7	5.0 ± 0.0	34.0 ± 0.7
PP65–35	11.0 ± 0.0	4.0 ± 0.0	5.0 ± 0.0	14.0 ± 0.0	4.0 ± 0.0	5.5 ± 0.7	31.0 ± 1.1
PP90–10	11 ± 0.0	3.5 ± 0.7	5.0 ± 0.0	12.5 ± 0.7	4.0 ± 0.0	5.0 ± 0.0	46.0 ± 1.1

Regardless of composition, all the Printlets sorbed some liquid from the medium, ranging from 11.0 ± 2.3% (PW35–65 formulation) to 46.0 ± 1.1% (PP90–10 formulation). Comparing amongst PW Printlets, PW65–35 absorbed the largest percentage of water. Contrastingly, for PP Printlets, PP90–10 was the formulation that exhibited the highest water uptake. In addition, PP Printlets generally sorbed more water than the equivalent PW Printlets (i.e. PP90–10 sorbed more water than PW90–10), with the exception of 65–35 formulations where the water uptake was similar. The water absorption differences could not be explained based on the degree of crosslinking. In general, the higher the degree of crosslinking, the lower the capacity to sorb solvent from the medium. The density of crosslinking depends on multiple factors, including the composition and duration of UV exposure during printing. According to exposure times (Table 2), the longest exposure time to print the torus was for PP90–10 Printlets (32 s); however, PP90–10 showed the highest water uptake. While a longer exposure time does not necessarily imply a higher degree of crosslinking, PP90–10 also contains the lowest relative concentration of non-reactive diluent. Therefore, it is expected that PP90–10 would have a greater degree of crosslinking compared to other formulations.

#### 3.2.6 In vitro release studies

**Fig. 11:**
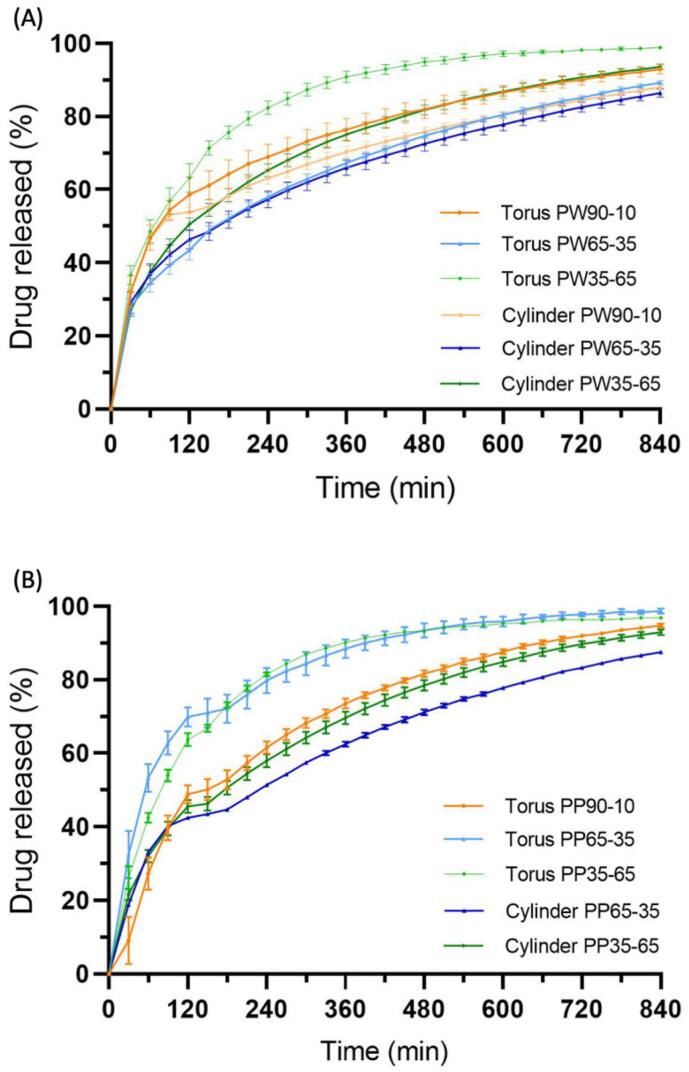
Paracetamol dissolution profiles from (A) PEGDA 575–Water Printlets and (B) PEGDA 700-PEG 300 Printlets.

Paracetamol release profiles from the Printlets are shown in Fig. 11. The release of paracetamol began during the gastric phase (pH 1.2) corresponding to the first two hours of the study. The paracetamol release was not affected by the subsequent change in pH (the new pH value was 6.8, simulating intestinal conditions). It was observed that 90% of the paracetamol was released in a period between 6 and 14 h from the torus shapes and between 12 and 15 h from the cylinder shapes. Therefore, drug release was faster for the torus shape than for the cylinder shape (Goyanes et al., 2015). This was expected as the torus shape yield a higher surface area to volume ratio (1.00) compared to the cylinder shape (0.49), allowing for a faster rate of drug release. All Printlets exhibited a sustained drug release profile and were insoluble in the dissolution media, suggesting that Printlets behaved similarly to inert matrix tablets.

The differences in the release profile observed between formulations were attributed to varying ratios of monomers and diluents. The presence of diluents decreased the cross-linking density of the matrices, permitting greater molecular mobility and faster drug diffusion out of the solid matrix and into the liquid dissolution media (Wang et al., 2016). Thus, for the PW35–65 and PP35–65 formulations, 90% drug release was achieved the fastest compared to the other PW (Fig. 11A) and PP (Fig. 11B) formulations with higher proportions of monomers.

Volumetric 3D printing using the rotary system was successfully implemented to produce torus and cylinder shaped Printlets containing paracetamol as the model drug. In each printing process, whose time ranged between 12.5 and 32 s depending on the formulation and shape, two objects were obtained simultaneously for the first time, halving the production times per unit to 6.25 and 16 s. These printing speeds were faster than those obtained in our previous study using a “mirror-based” volumetric printer (Rodríguez-Pombo et al., 2022). Admittedly, printing parameters such as the rotation speed and exposure time must be re-optimized for different formulations, including changes to the resin composition, the drug used, and the drug load. However, as demonstrated in our numerous previous studies involving vat photopolymerization-based 3DP techniques, printing dosage forms with this technology and with higher drug content should remain feasible (Vivero-Lopez et al., 2021; Xu et al., 2021a; Xu et al., 2021b). The key aspect to take into account is the transparency of the resin so that the projection of light is not scattered by the turbidity. Therefore, the technology reported in this article is one of the fastest for the manufacture of personalised medicines, allowing batch production of customised medicines tailored to individual patients.

While the present study only explores the production of two objects simultaneously, production of larger batches using rotary volumetric printing is possible given a larger resin container and larger light projection. The container used in this study was only large enough to permit the printing of two objects. Therefore, by augmenting the size of the resin container, printing time per object can be significantly shortened, making this novel 3D printing technique also potentially suitable for the mass manufacturing of medicines.

## 4 Conclusions

Rotary volumetric printing was successfully used, for the first time, to simultaneously produce two paracetamol-loaded torus-shaped and cylindrical tablets within 12 to 32 s, depending on the formulation and shape. These printing times were shorter than any previous study on pharmaceutical 3D printing, thus representing to date the fastest means of manufacturing personalised 3D printed drugs. Drug release studies demonstrated the possibility of modifying the Printlets' drug release profile by varying the ratio of photoreactive monomers and diluents. This study also demonstrated the successful production of two objects using a volumetric printer within a single print. By increasing the size of the resin container, larger batches of objects may be printed simultaneously. The versatility of rotary volumetric 3D printing therefore facilitates its possible deployment for both mass and personalised manufacturing of medicines. With further optimization, such as mitigating the inconsistent refraction of light, rotary volumetric printing could become a pivotal technology for medicines manufacturing in industry and at the point-of-care.

The following are the supplementary data related to this article.

Supplementary video 1 Video showing volumetric printing of two torus simultaneously using PW35-65 resin Supplementary video 1

## CRediT authorship contribution statement

**Lucía Rodríguez-Pombo:** Data curation, Formal analysis, Investigation, Methodology, Writing – original draft, Writing – review & editing. **Laura Martínez-Castro:** Data curation, Formal analysis, Investigation, Methodology, Writing – original draft, Writing – review & editing. **Xiaoyan Xu:** Data curation, Formal analysis, Investigation, Methodology, Writing – original draft, Writing – review & editing. **Jun Jie Ong:** Data curation, Formal analysis, Investigation, Methodology, Writing – original draft, Writing – review & editing. **Carlos Rial:** Software, Validation. **Daniel Nieto García:** Conceptualization, Resources, Validation, Writing – review & editing. **Alejandro González-Santos:** Data curation, Formal analysis, Investigation. **Julian Flores-González:** Supervision, Writing – review & editing. **Carmen Alvarez-Lorenzo:** Supervision, Writing – review & editing. **Abdul W. Basit:** Conceptualization, Resources, Supervision, Writing – review & editing. **Alvaro Goyanes:** Conceptualization, Methodology, Project administration, Resources, Supervision, Writing – review & editing.

## Declaration of Competing Interest

The authors declare that they have no known competing financial interests or personal relationships that could have appeared to influence the work reported in this paper.

## Data Availability

Data will be made available on request.
